# Supplementary material for: Are carbon emissions trading and green financial instruments synergistic? -Comprehensive quantitative research based on content analysis
Source: PLoS One. 2024 Mar 7;19(3):e0298601. doi: 10.1371/journal.pone.0298601 (PMC10919601; doi:10.1371/journal.pone.0298601)
Supplement: S1 Data — (ZIP) [file pone.0298601.s001.zip › Data/地级市Z_F工作报告.zip!/╕ú╜¿/─■╡┬╩╨/└┤╫╘╣1⁄2╓┌║┼í╛┬φ┐╦╩2╛▌═°í┐_2011.docx]

各位代表：

现在，我代表市人民政府向大会作政府工作报告，请各位代表连同《宁德市国民经济和社会发展第十二个五年规划纲要（草案）》一并审议，并请市政协各位委员和其他列席人员提出意见。

一、2010年和“十一五”期间经济社会发展回顾

2010年，在省委、省政府和市委的正确领导下，我市各级政府坚持以科学发展观为指导，认真贯彻十七届三中、四中、五中全会以及省委八届八次、九次和十次全会精神，按照市委的决策部署，集中精力实施“五大战役”，全力推动宁德科学发展、跨越发展，经济社会发展取得新的更好成效。全市生产总值736亿元，增长15%；财政总收入62.8亿元，增长40.6%，其中，地方级财政收入40.5亿元，增长47.1%；全社会固定资产投资371.7亿元，增长29.4%；外贸出口总额9.74亿美元，增长39.3%；验资口径实际利用外商直接投资0.71亿美元，增长25.1%；本外币各项存款余额673亿元、贷款余额722亿元，分别增长37.9%和26.4%；社会消费品零售总额233.5亿元，增长16.1%；居民消费价格总水平上涨3.8%；城镇居民人均可支配收入16815元，增长11.0%；农民人均纯收入6542元，增长12.1%；城镇登记失业率3%；人口自然增长率6.5‰。单位生产总值能耗降低0.7%，二氧化硫排放量、化学需氧量排放减少基本达到省定目标。

一年来，主要工作和成效是：

（一）积极把握发展机遇，环三战略加快推进

过去一年，是宁德在新的起点上加快推进环三都澳区域发展的一年。胡锦涛总书记等中央领导到福建考察指导，习近平副主席、王兆国副委员长等党和国家领导以及省委、省政府主要领导先后到宁德考察调研，充分体现了中央和省委、省政府对宁德工作的关心和支持。省委八届九次全会作出“推动福建科学发展、跨越发展”的决策部署，提出“先行先试、加快转变、民生优先、党建科学”的总体要求；市委二届十一次全会审议通过的制定“十二五”规划建议，明确未来五年发展的指导思想、奋斗目标、主要任务和重大举措。全市上下全面落实省委、省政府的工作部署，按照市委的要求，集中力量大干150天、打好“五大战役”，凝心聚力、真抓实干，掀起了比学赶超、谋求跨越的热潮，环三集聚效应进一步显现。在福建省与央企项目合作洽谈会和我市与央企（名企）签约仪式上，共签订项目20项、总投资2322.6亿元，项目总数和投资额均居全省前列；首届海峡两岸电机电器博览会和第十一届宁德投洽会，共签约项目165项，总投资达336亿元，其中：环三都澳区域项目54个，投资额246亿元。

（二）加快发展方式转变，经济持续较快增长

1、现代农业稳步发展。去年农林牧渔业总产值236亿元，增长4.4%。粮播面积和粮食产量保持稳定，茶叶、水产、食用菌等主导产业不断发展壮大。农民增收“八大工程”扎实推进，“六大能力”进一步提升。新增国家级农业标准化示范区6个，新增产值超亿元农业龙头企业2家，完成国家农业综合开发土地治理3.5万亩，硬化农村公路500公里，除险加固海堤29公里，加固病险水库39座，解决15.8万人农村人口饮水安全问题，完成9个乡镇360个村垃圾治理，新建农村户用沼气池3890口。“五新”技术推广、院市合作项目、农村实用技术远程培训等农业科技工作全面提升，完成农村实用技术远程培训6万人次。农民专业合作社得到发展。海上养殖业综合整治成效明显。林权配套改革不断深化，全年完成造林任务26.2万亩。村级公益事业建设“一事一议”财政奖补试点工作顺利推进。防灾减灾工作扎实有效。

2、产业升级步伐加快。去年实施27个产业振兴项目、13个成长型工业新增长点项目和21个重点工业技改项目，新增规模以上企业180家，全年规模以上工业总产值917.6亿元、增加值232.9亿元，分别增长42.6%和32.5%，增幅连续6年居全省首位。电机电器、船舶修造、食品、电力等传统产业产值分别达143.4亿元、44.7亿元、131.5亿元和94亿元。宁德核电一期累计完成投资超200亿元，聚合物锂离子电池、寿宁高纯锆等项目即将建成投产。新认定高新技术企业7家、省级企业技术研究中心4个，成功对接“6?18”项目435项、总投资67.8亿元。40家担保机构提供担保贷款超300亿元。现代服务业加快发展，第三产业增加值281亿元，增长8.7%。以屏南白水洋、福鼎太姥山和福安白云山为核心景区的宁德地质公园成功申请列入世界地质公园网络名录，屏南县顺利通过省级优秀旅游县验收，全市共接待游客701万人次、旅游收入53.6亿元，分别增长13.2%和15.5%。

3、对外开放持续拓展。积极组织和参与招商活动，全年共签约项目174个、投资总额753.8亿元，新批外商投资项目18项、合同外资2.9亿美元。成功举办香港?宁德投资洽谈会和第二届海峡论坛两岸少数民族联谊交流会，积极参加“9?8”、“5?18”等大型招商活动，多次组团赴台开展经贸文化交流活动，全年签约外资项目47项、总投资28亿美元。对外贸易快速回升，进出口保持强劲增长，进口总值2.36亿美元，增长200.8%，增幅居全省首位。福建省电机出口基地建设加快推进。与美国哥伦布市缔结友好城市。

4、环境保护力度加大。节能减排责任制和环保监管“一岗双责”制度有效落实。福安、柘荣、古田、周宁、屏南、寿宁污水处理厂相继投入运行，开工建设宁德北区污水处理厂、福鼎第二污水处理厂；完成市生活垃圾焚烧发电厂主体工程，建成周宁、寿宁、柘荣垃圾处理场。 “两江三溪”重点流域水环境综合整治扎实推进，境内主要流域水质功能达标率和饮用水源水质达标率达100%，敖江流域古田段水质达到省定指标。海洋环境保护得到加强。土地、矿产资源开发整合和保护力度加大，连续12年实现耕地占补平衡。“四绿”工程启动实施，森林覆盖率继续保持全省沿海设区市前列。

（三）抓住重点着力突破，“五大战役”成效明显

1、重点项目建设加速推进。118个重点项目完成投资199.6亿元，增长27.8%，占年度计划106%。宁德核电一期、宁武高速公路宁德段、宁古二级公路等项目建设进展顺利，霞浦大京风电场、宁德保障性住房金涵小区一期工程、福安东元电机等建成投产，全年竣工项目32个。合福铁路宁德段、白马港铁路支线、京台高速公路宁德段、沈海高速复线漳湾至连江浦口段等开工建设，全年新开工项目55个。衢宁铁路宁德段、海西高速公路网古田至屏南连接线、宁德核电二期、大唐国际宁德综合储运等项目前期工作取得突破。

2、新增长区域发展扎实推进。151项新增长区域项目完成投资160亿元，占年度计划的106.9%。海西宁德工业区完成总体规划编制并通过省政府审批，霞浦长腰岛征海征地工作开始启动，1000万方商业储油等“四个一”项目前期工作扎实推进。漳湾临海工业区中科院中国宁德新城镇综合改革示范区一期、火箭研究院风机制造基地奠基开工，福建（宁德）微软技术中心、中船九院宁德分公司挂牌开业，安发生物科技园一期投产；鞍钢宁德大型钢铁项目取得实质性突破，我市与鞍钢集团正式签订投资协议。赛江临港工业区鑫茂冷轧硅钢一期、鼎信镍合金一期等项目建成投产，海和20万吨高镍合金等项目前期工作有序推进。沙埕临港工业区江南造船项目进展顺利，福鼎福丰30万吨镍合金项目启动建设。山区重点工业片区古田天宝矿业集团技改扩建、屏南榕屏化工公司10万吨双氧水、周宁抽水蓄能电站、寿宁三祥新材料、柘荣三本油茶等项目建成投产或按序时进度推进。

3、城市建设强力推进。编制完成环三城镇群规划、城市总体规划修编方案、滨海新城概念性规划及溪南城市组团、赛江城市组团、龙安城市组团规划，福安、福鼎、霞浦城市规划展示馆启动建设。中心市区环城北路改造完成，国宝路南段竣工，中心市区和福安、福鼎、屏南、霞浦城区主干道沥青化、电线下地工程启动建设，街道路面铺装率达100%。新增城市建成区绿地面积61公顷，中心市区和福安、福鼎道路绿化普及率和达标率分别超过95%和80%。寿宁县城东区一期等项目建成试运行，东方伟业广场改扩建主体工程完成，宁德万达广场开工建设。宁德、福鼎被命名为“省级园林城市”。

4、小城镇改革发展加快推进。福安赛岐、福鼎秦屿2个省级试点镇、17个市级试点镇建设加快推进，全年完成投资21亿元。试点乡镇总体规划、专项规划和分区控制性详规编制全面完成，赛岐中心小学扩建、秦屿镇文化中心等一批项目扎实推进。实施农村土地整治和城乡建设用地增减挂钩，完成400亩土地整治项目和增减挂钩指标使用。

5、民生工程有力推进。20件31项为民办实事项目全面完成。建成宁德师院新校区一二期工程、宁德师院附小和体育中心二期工程等一批项目，开工建设宁德市医院迁建工程、宁德职业技术学院、市委党校、市社会福利中心、市妇女儿童活动中心、市图书馆等项目。加固重建中小学校舍24.55万平方米，新建农村寄宿制学校建筑面积3.44万平方米，完成中小学布局项目329个。改造提升县级医院4所、乡镇卫生院14所、社区卫生服务中心4所。建成保障性住房2521套。新建农村敬老院17所，完成农村五保“幸福园”建设1000户。新建乡镇综合文化站21个、乡镇（街道）青少年校外体育活动场所89个、乡（镇）农民体育健身活动中心9个、行政村农民体育健身工程283个。建成菜市场等商业网点17个、村级综合服务场所776个、县乡村三级救灾避难场所39个，新建或改建公厕31座。全面完成以古田县为重点的农村住房灾后恢复重建任务。

（四）统筹发展社会事业，社会保持和谐稳定

1、社会保障逐步完善。全年新增城镇就业2.6万人、下岗失业人员再就业1.02万人、农村劳动力转移就业4.6万人。欠薪应急保障金制度全面建立，劳动争议结案率保持在95%以上。医疗、工伤、生育保险实现设区市统筹，未参保集体企业退休人员基本养老保障和其他社会保障遗留问题基本解决。城镇居民医保、新农合筹资水平和补助标准提高，新型农村社会养老保险试点扎实推进。“金保工程”和社会保障卡项目建设全面启动。

2、社会事业不断进步。义务教育逐步均衡发展，标准化学校建设取得实效，高中课改有序推进，学前教育得到加强，古田县通过省级“双高普九”验收。宁德师专升格为宁德师范学院，宁德电大和业余大学、闽东高级技工学校和工业学校整合重组顺利完成。文化产业推介和文化遗产保护力度加大，文学艺术、新闻出版等事业进一步繁荣，“中国水密隔舱福船制造技艺”被联合国批准列入急需保护的非物质文化遗产名录，“柘荣剪纸”入选联合国教科文组织人类非物质文化遗产代表作名录。隆重举办撤地建市十周年系列文化活动。体育事业蓬勃发展，蕉城籍运动员李雪艳在第16届广州亚运会上获得2枚金牌。保持稳定低生育水平，连续17年完成省下达的人口控制目标。第六次全国人口普查工作顺利开展。对口支援工作成效显著，援建彭州升平镇项目全面完成。人事、编制、统计、老区、库区、民族、宗教等工作取得新成效，档案、老龄、地方志、农机、水文、气象、残疾人事业、关心下一代等工作进一步加强。

3、社会管理得到加强。认真落实社会矛盾化解、社会管理创新和公正廉洁执法三项重点工作，新一轮平安创建活动深入扎实，人民群众对社会治安满意率达94.8%。学校及周边安全稳定措施有效落实，全市中小学、幼儿园全部配备保安人员。安全生产“一岗双责”全面落实，重点行业、重点领域安全生产专项整治成效明显，应对突发公共事件能力逐步提升。殡葬改革稳步推进，全市平均火化率和专项治理乱建坟墓工作取得实质性进展。国防动员、民兵预备役、双拥支前工作取得新成绩，人防、海防、反走私等工作取得新进展。

（五）依法行政水平稳步提高，政府自身建设得到加强

各级政府依法接受人大及其常委会法律监督和工作监督，依法执行人大及其常委会的决定决议，坚持向人大及其常委会报告工作制度，高度重视人大常委会听取专项工作报告、开展执法检查等审议意见，认真办理人大代表建议、批评和意见。支持人民政协履行政治协商、民主监督、参政议政职能，认真办理政协提案，积极采纳政协调研视察成果。全年共办理市人大代表建议221件、市政协提案308件，办结率均为100%。广泛听取各民主党派、工商联、各人民团体、无党派人士及社会各界人士意见。 “五五”普法顺利完成。推进政务公开和政府信息公开，深化效能建设、绩效管理，加大治庸治懒力度，推行“容缺预审”和“并联审批”，行政效率进一步提高。全面规范行政权力运行，大力推进规范行政处罚自由裁量权工作，做好行政复议工作，加强重点领域、重点项目审计和稽察，纠正损害群众利益的不正之风。认真落实领导干部廉洁自律各项规定，反腐倡廉工作不断加强。

各位代表，2010年工作任务的完成，标志着市一届人大八次会议通过的“十一五”规划基本完成，宁德经济社会发展进入新的发展阶段，为推进科学发展、跨越发展积蓄了能量、奠定了基础。一是综合实力显著增强，跨入沿海快速发展行列。全市生产总值和人均生产总值比2005年翻一番，财政总收入是2005年的3.1倍。全市GDP年均增长13.9%，高于“十一五”规划1.9个百分点；2010年人均GDP达2.4万元，超过“十一五”规划目标，年均增长14.1%。财政总收入和地方级财政收入年均分别增长25.3%、26.2%，高于“十一五”规划目标12.3和13.2个百分点。二是产业结构更趋优化，成为海西重要增长极。全市三次产业比例由2005年的23.2:34.7:42.1调整为2010年的18.5:43.4:38.1。现代农业稳步发展，茶叶、水产、食用菌三大主导产业产值占农林牧渔总产值的58%。工业总产值突破1000亿元，增加值264亿元，分别比上年增长37.5%和27.8%。电机电器产业入选中国百佳产业集群，福鼎荣获“全国化油器名城”称号。服务业增加值年均增长12.9%，比“十五”提高1.3个百分点。中国驰名商标实现零的突破，总数达8个。三是基础设施不断完善，投资环境进一步提升。全社会固定资产投资五年累计突破1200亿元，是“十五”的2.7倍。温福铁路建成通车，宁德大唐火电厂一、二期等一批重点工程相继建成投产，宁德核电一期、宁武高速公路等一批重大项目开工建设。全市新增高速铁路里程160公里、港口吞吐量626万吨、电力装机容量280万千瓦。水利“六千”工程、农村“年万里”路网工程提前完成，防灾减灾体系进一步健全。四是城乡面貌焕然一新，绿色宜居城市雏形初显。中心城市功能日趋完善，五年累计投入100多亿元，建成宁德会展中心、城市规划展示馆、东湖南北岸公园等市政地标项目以及一批道路、景观等城市配套基础设施，中心城区建成区面积从2005年的11.8平方公里扩大至23平方公里。城镇化进程加快推进，城镇化率从2005年的38.2%提高到41.5%。五是改革开放富有成效，经济发展活力增强。农村综合改革扎实推进，国有资产监督管理体系逐步完善，财政、流通、城市管理以及教育、科技、文化等领域的体制改革不断深化。招商引资力度加大，五年累计签约内资项目801项，总投资4306.8亿元，其中对接央企项目28项，总投资2986.6亿元；外资项目186项，总投资50.6亿美元。外贸出口快速增长，五年累计出口35亿美元，是“十五”的3.4倍。宁台交流合作更加密切，与台湾基隆市缔结友好城市，对台经贸合作、文化交流、民间往来都取得新拓展。六是社会事业稳步发展，公共服务水平逐步提升。全市财政用于社会事业等方面支出五年累计106亿元。科技创新步伐加快，高新技术产业增加值占工业增加值的比重逐步提高。各类教育朝均衡方向发展，义务教育各项指标有长足进步，高中阶段毛入学率提高8个百分点，中职在校生超过4万人。医药卫生体制改革扎实推进，公共卫生服务体系逐步完善，重大疾病防控和卫生应急处置能力继续增强。覆盖城乡的公共文化服务网络不断完善，公益性文化事业加快发展，群众文化生活更加丰富。环境保护持续加强，实现每县有一个污水处理厂和垃圾处理场，处理率分别达到60%和70%，生态环境质量继续位居全省前列。荣获“全省双拥模范城”三连冠和“全国双拥模范城”二连冠。七是人民生活水平稳步提高，小康社会建设迈出新步伐。城镇居民人均可支配收入和农民人均纯收入分别年均增长11.7%和11.4%。城镇登记失业率控制在4%以内。以社会保险、社会救助、社会福利为基础的社会保障体系逐步完善。 “造福工程”及滑坡险情村搬迁实施四年来，累计完成搬迁9.5万人。第一轮158个新农村试点示范村建设扎实有效，第二轮278个县级以上扶贫开发重点村整村推进圆满结束。

回顾过去一年的工作，总结过去五年的实践，我们深深体会到，要实现宁德经济社会又好又快发展，必须始终坚持以科学发展观统领全局，全力实施环三都澳区域发展战略，加快建设海峡西岸经济区东北翼中心城市；必须始终坚持项目带动、品牌带动，以项目带动主导发展，以品牌带动提升发展，持续壮大经济总体实力；必须始终坚持改革创新、扩大开放，在深化改革中增强发展的活力和动力，在扩大开放中赢得发展的机遇和空间；必须始终坚持着力民生、服务百姓，多做惠及群众的好事实事，让广大人民群众共享改革发展成果；必须始终坚持依法行政、科学理政，转变政府职能，优化发展环境，改进工作作风，不断提高各级政府的执行力。

“十一五”发展的成就来之不易，这是市委正确领导、科学决策，全市人民齐心协力、奋力拼搏，社会各界人士广泛参与、大力支持的结果。在此，我代表宁德市人民政府，向全市人民致以崇高的敬意，向给予政府工作有力支持和有效监督的人大代表、政协委员、各民主党派、工商联、各人民团体、无党派人士、离退休老同志和社会各界人士，向为宁德发展作出积极贡献的中央及省属驻宁单位、驻宁部队、武警官兵、公安民警、民兵预备役人员，向关心支持宁德发展的港澳台同胞、海外侨胞、国际友人，表示诚挚的感谢！

在肯定成绩的同时，我们清醒认识到发展中还面临不少困难和问题，主要是：经济总量小，总体实力弱，在全省位次仍然靠后；产业结构不合理，企业市场竞争力和创新能力不强；项目总数较多，但在谈多、落地少；项目要素保障压力大，资源环境约束增强；消费环境有待改善，内需支撑要素不足；财政金融压力大，对投资需求产生较大制约；干部作风还有待进一步改进，一些单位、个别干部办事效率低下、服务质量不高，甚至存在不作为、乱作为和消极腐败现象。此外，城镇化率、化学需氧量排放量、国民平均受教育年限等未达到“十一五”预期目标，社会消费品零售总额增幅未达到2010年确定的目标。对这些问题，我们要深入分析原因，逐项研究对策，采取更加有力的措施切实加以解决。

二、“十二五”时期主要目标任务

“十二五”时期是宁德加快转变、跨越发展的重要战略机遇期。从外部环境看，国家鼓励东部地区率先发展、支持海峡西岸经济区在两岸交流合作中发挥先行先试作用；我市沿海列入国家主体功能区重点开发区域，环三都澳区域列入省十大新增长区域首位；国家鼓励经济结构战略性调整，重化产业加快向沿海深水港区转移集聚，为我们挖掘潜力、发挥优势，全力推进跨越发展创造了有利条件。从宁德自身看，经过多年努力，综合实力明显提升，基础设施更加完善，区位、港口、生态、对台等优势进一步显现，积蓄了跨越发展的强大动力。但宁德还存在发展环境、发展后劲和要素支撑不足等问题。我们一定要奋力追赶，以“拼”和“闯”的努力，勇于应对挑战，敢于开拓创新，全力推动科学发展、跨越发展，力求发展有更大作为、人民福祉有更大改善。

根据市委二届十一次全会通过的《中共宁德市委关于制定宁德市国民经济和社会发展第十二个五年规划的建议》，在综合考虑未来国内外发展趋势和市情实际的基础上，《规划（草案）》确定了今后五年经济与社会发展的目标重点：

（一）发展速度上。“十二五”期间，我市生产总值要在“十一五”年均增长13.9%的基础上，抓住机遇，加快发展。在认真研判国内外经济形势、综合考虑各种要素支撑的基础上，经过反复测算和研究讨论制定了“十二五”经济社会发展的主要目标：地区生产总值年均增长16%以上，财政总收入年均增长25%左右，全市经济发展水平与全省平均水平差距明显缩小，在海西发展格局中位次前移。这个目标比全省10%的增长高6个百分点，既考虑了实际基础和发展潜力，也体现了跨越发展的内在要求和紧迫感、责任感，是好中求进、进中求快的目标。只要我们铁心拼搏、爬坡攻坚，大干快上、奋力赶超，是有可能在新的起点上实现新目标的。

（二）结构调整上。“十二五”期间，要加快产业结构调整，推动全市经济由传统产业构成为主向新兴产业、先进制造业、现代农业和现代服务业协调发展转变。产业结构调整关键靠项目、靠投入、靠技术创新。要加快农业发展方式转变，大力扶持茶叶、水产、食用菌等主导产业，增强保障供应能力、市场竞争能力和抵御风险能力。要把工业的着力点和突破口放在发展临海重化产业、培育战略性新兴产业和改造提升传统产业上，加快推进一批符合国家产业政策的临海石化、大型钢铁、先进机械制造以及新能源、新材料、生物医药等项目建设，不断提升产业集聚度和竞争力。要加快发展现代服务业，促进消费转型升级，增加服务业在国民经济中的比重，提高消费对经济增长的贡献率。

（三）城乡建设上。“十二五”期间，要在环三规划指导下，以同城化为目标，推进周边区域围绕中心城区来拼图规划。近期要按照“临海、跨海、环海”的发展步骤，打破行政区划，统筹环三都澳区域，科学规划宁德组合城市，组织实施滨海新城拓展工程、重点片区提升工程、标志景观打造工程，加快推进中心城市临海布局、环湾发展，不断提升中心城市的辐射带动能力；远期要按照“港口、产业、城市、生态”一体化要求，优化资源配置，合理布局，做大做强中心城市，逐步形成 “一市多区多组团”的绿色宜居海湾新城。同时，要以县城为中心，立足现有基础，力争用五年左右时间培育形成各自的县域优势产业和特色产业，促进一批具有交通、产业、人口优势的中心集镇建设成为农村区域性经济文化中心，使县域经济总量、产业结构和城镇发展提高到新的水平。

（四）改革开放上。“十二五”期间，要以更大决心和力度推进重点领域和关键环节改革，重点在深化行政管理、财政金融、社会事业等方面体制改革上下功夫，切实增强发展动力和活力。要改进工作，强力推进南与福州、泉州和珠三角，北与温州、长三角，西与中部内陆地区，东与台湾的区域交流合作，在扩大开放、深化合作中引进更多资金、技术、管理和项目，争取更多的外来资源培育新的发展优势。

（五）民生发展上。“十二五”期间，要更加顺应人民的新期待、新要求，把保障和改善民生作为一切工作的出发点和落脚点，像抓经济建设一样重视民生工程、抓好民生事业，将更多的人力、财力、物力投入民生领域，加快发展各项社会事业，完善保障和改善民生的制度安排，推进基本公共服务均等化，加大收入分配调节力度，坚定不移走包容性增长和共同富裕的道路，使经济发展的成果更多更好地惠及全市人民，实现社会的公平正义，不断提高人民群众幸福指数。

三、2011年政府工作主要安排

2011年是建党九十周年，也是实施“十二五”规划的开局之年。我们要全面贯彻落实党的十七大、十七届三中、四中、五中全会和省委八届九次、十次全会以及中央、全省经济工作会议精神，以邓小平理论和“三个代表”重要思想为指导，深入贯彻落实科学发展观，以科学发展为主题，以转变方式为主线，按照市委二届十一次全会的决策部署，抢抓机遇，乘势而上，聚精会神，心无旁骛推进环三战略实施、推动宁德跨越发展，着力促进增长、调整结构，统筹城乡、惠顾民生，深化改革、扩大开放，开展“五个年”活动，打好“五大战役”，大干开局之年，实现经济持续快速发展和社会和谐稳定。

根据省委八届十次和市委二届十一次全会精神，市委常委会研究提出，今年经济社会发展的主要预期目标是：地区生产总值增长15%以上，实际工作按16%安排；全社会固定资产投资增长25%；社会消费品零售总额增长15%；出口总值增长15%以上，验资口径实际利用外商直接投资增长10%；地方财政收入增长16%以上；城镇居民人均可支配收入增长12%以上;农民人均纯收入增长12%以上；居民消费价格总水平涨幅控制在4%左右；城镇登记失业率控制在4.2%以内；人口自然增长率控制在7‰以内；单位生产总值能耗、化学需氧量、氨氮、二氧化硫、氮氧化物年度排放量控制在省定目标内。

实现上述目标，必须扎实做好以下五个方面工作：

（一）发展大产业，不断壮大经济实力

1、加快推进现代农业建设步伐。认真落实强农惠农政策，开展粮食作物品种改良、新品种开发、综合配套技术推广等粮食高产创建活动，建立6个万亩示范片区，加强粮食和应急种子储备管理，确保粮播面积和产量稳定。围绕茶叶、水产、食用菌等主导产业和林竹、畜禽、果蔬等特色产业，积极拓展特色高效农业基地规模，大力推进农业产业化，力争在各县（市、区）建设1-2个现代农业加工园区（含茶业产业园），研究推广新技术，发展特色水果和蔬菜。支持发展和规范农民合作社和农村行业协会，推进农业“五新”入户，大力开拓农产品市场。着力培植和扶持一批省、市级农业产业化龙头企业，力争新增产值超亿元企业2家以上。改善以水利为重点的农村基础设施条件，实施国家农业综合开发土地治理2.5万亩，完成海堤除险加固21公里、小型水库除险加固11座，解决10万以上农村人口安全饮水问题，实施新一轮农村电网改造升级，新建渔港33个，开工建设三沙国家级中心渔港，推进农村危房改造、农村信息化和电气化，促进基础设施向农村延伸。开展农村环境综合整治，以农村户用沼气池建设带动改厨、改厕、改圈，发展农业循环经济。开工建设新一代天气雷达，完善动植物疫病防控体系，提高重大灾害预警预报、快速反应和紧急救援能力。继续推进社会主义新农村试点示范村建设，扎实推进第三轮“整村推进”扶贫开发重点村工作。

2、加快推进新型工业化进程。发挥我市海洋资源的优势，推进海洋经济发展。石化产业要推进中海油海西宁德工业区建设，完成霞浦溪南长腰岛及周边区域征海征地工作，全力推进1000万方商业储油和300万吨/年LNG及综合利用、3000吨级工作船码头、环绕长腰岛储油配套道路、福州至福鼎的LNG输气管线等“四个一”项目建设，深化LNG接收站、燃气发电厂、炼化一体化等项目前期工作。冶金产业要积极推进鞍钢宁德大型钢铁项目前期，全面启动项目配套基础设施和土地整理工作；加快福安、福鼎、寿宁、周宁镍合金以及冷轧硅钢和台湾烨联钢铁60万吨镍合金、福安300万吨不锈钢生产项目建设。机械装备制造业要推进白马船舶工业园、福鼎江南船厂、蕉城豪华游艇、福安立松大型精密铸件等项目建设，积极引进央企、外企提升船舶制造企业，加快推进火箭研究院大型风机制造基地建设。能源产业要加快宁德核电一期工程建设，推进大唐火电二期商业运行审批和三期建设工作，深化宁德核电二期、华能霞浦核电、周宁抽水蓄能发电等项目前期工作；加快推进蕉城、福鼎两个锂离子电池项目和福安博瑞特车用锂离子电池、霞浦吉阳太阳能电池、福鼎聚晶光学太阳能项目建设。新材料产业以寿宁三祥公司为龙头，大力发展锆系列高新产品，加快争创国家实验室和企业上市步伐。生物与新医药产业要积极支持海西药城、安发（福建）生物科技园和周宁无患子生物开发项目建设。同时，以正在实施的龙头项目为抓手，做好项目规划对接，在石化、冶金、机械、船舶、食品加工等5个方面做长做大做足产业链，推进电机电器、汽摩配件等传统产业主导产品升级换代并向上下游产业延伸。

3、加快推进现代服务业发展。以宁德世界地质公园申报成功为契机，优化闽东北亲水游线路，大力推进白水洋、太姥山5A级景区创建和鲤鱼溪、九龙际、白云山4A级景区的创建工作，组建市旅游开发公司，加强与周边城市及台港澳地区的旅游协作和线路对接，发展壮大旅游产业。扶持现代物流业，依托港区、园区、城区和道口，规划建设现代物流园区和物流中心，培育和引进一批覆盖范围大、功能作用强的专业化骨干物流企业。关注房地产政策的新变化，有序引导房地产市场开发和市民消费。落实“菜篮子”市长负责制，继续实施“万村千乡”市场工程，改造提升城乡集贸市场和批发市场，实施家电、摩托车下乡和汽车以旧换新，重点扶持12个集贸市场、252个农家店、60个社区便利店、30个菜市场等商业网点建设。切实把稳定物价总水平放在突出位置，加强生活必需品市场供求调节和价格调控，保障消费者合法权益。

（二）建设大交通，有力支撑跨越发展

1、建设环三交通网络。组织“交通建设会战年”，加强以港口为取向的陆海空立体交通网络建设，重点抓好向中西部横向拓展的铁路、高速公路建设，以路带港，以港兴市，港城联动，跨越发展。今年着力完善高速公路网，加快建设宁武高速公路宁德段、京台高速公路宁德段、沈海高速复线漳湾至连江浦口段，开工建设沈海高速复线福鼎至漳湾段，力争开工建设海西高速公路网古田至屏南连接线、福安至寿宁高速公路，深化宁德至古田高速公路前期工作。着力构筑区域性铁路网，加快建设合福铁路宁德段，开工建设衢宁铁路宁德段，全面完成温福铁路宁德段配套建设，启动宁德至古田铁路规划研究，深化宁漳高铁、沿海铁路货专前期工作。着力推进国省干道建设，建成宁古二级公路、省道301线寿宁城关至大熟段和霞浦城关至福安溪柄段、省道302线周宁浦源至政和界段、省道202线屏南城关至梅花地段，加快推进省道202线寿宁城关至政和界段、省道303线屏南城关至建瓯界段，开工建设省道202线北村至白水洋段，推进省道201线宁德城关过境段（宁德滨海大道）前期工作，改造三级以上公路里程178公里。着力突破空港设施，开工建设宁德霞浦机场民航扩建工程。

2、构建港口集疏运系统。着力推进港口码头开发，推进城澳作业区1#、2#泊位建设，开工建设漳湾对台客货码头和8#、9#5万吨码头、白马作业区14#泊位工程、福建大唐国际宁德综合储运一期工程、福鼎沙埕湾5万吨码头等项目。着力推进疏港通道建设，建成溪南疏港二级公路，加快建设白马港铁路支线，开工建设溪南疏港高速公路，深化溪南、漳湾、城澳等疏港铁路支线前期工作，提高港口通达性。

3、完善农村交通路网。继续推进农村公路基础网络改造、村通客车工程，改造危桥18座，撤渡建桥9座，重点做好县乡道改造工程，提升农村公路好路率，新增农村公路里程420公里，力争完成520公里。

（三）构建大城市，打造绿色宜居滨海新城

1、增强中心城市辐射带动能力。拓展城市发展空间，科学规划与调整行政区划，理顺市区两级财政、土地等体制，积极支持蕉城区壮大整体实力，加快滨海新区、铁基湾新城规划建设。加快万达广场城市综合体建设，全面推进“义大世界”城市综合体、汽车4S中心、红星美凯龙家具城、宁德列车乘务培训中心及研发平台等项目；开工建设福宁北路、金漳路、薛令之路南段、富春路。加强中心市区饮用水源地保护，加快第三水厂建设。完善闽东工业园区扩展规划，全力推进园区安征迁工作以及水、电、路、集中治污等配套设施建设，加快推进和引进一批产业项目。强化城市综合管理，下决心坚决清理查处“两违”行为，努力改善城市面貌，争创国家级园林城市和省级卫生城市、先进文明城市。

2、提升县域统筹发展能力。引导各县（市、区）立足各自资源、产业和区位优势，力争每个县（市、区）打造一个以上在全省有影响、有实力的特色产业。加强县域重点产业集中区建设，形成县域产业与区域产业的协作配套关系，发展壮大县域经济。做好旧城改造和新区建设，策划实施一批城市综合体、公共交通、社会事业项目，建设一批学校、医院、体育场馆、城市景观工程、保障性住房等功能性项目，进一步提高城市建设层次和品位。加快解决制约县域经济发展的体制机制、科技人才、基础设施、公共服务等突出问题，促进各类要素向城镇聚集，增强县域经济发展活力。

（四）实施大民生，提高群众幸福指数

1、建立健全社会保障体系。积极做好高校毕业生、农村富余劳动力和城镇就业困难人员的就业工作，确保城镇新增就业2.4万人，城镇登记失业人员再就业5000人，新增农村劳动力转移就业3万人。逐步扩大社会保险覆盖面，加快建设“金保工程”，全面完成249万张社会保障卡发放任务。进一步调整收入分配关系，扩大中等收入人群的比重，改善低收入家庭生活水平，引导企业建立健全职工工资正常增长机制。完善城乡低保动态管理机制，提高农村“五保”供养水平，加强城乡困难家庭专项救助和临时救助。改善优抚对象和革命“五老”人员待遇。发展社会福利、慈善和残疾人事业，维护妇女、儿童和少数民族合法权益。扎实开展第三批新型农村社会养老保险工作，完善被征地农民、征海渔民养老保障政策。实施“造福工程”及滑坡险情村搬迁2万人，建设保障性住房6.2万平方米，建成廉租房1313套，规划建设市本级保障性住房、安置房1484套。

2、加强生态环境保护。启动建立生态补偿机制，突出抓好江河源头区、重点水源涵养区、饮用水源区、防风固沙区、重点渔业水域的生态保护；着力推进“森林宁德”和生态市建设，积极实施“四绿”工程，扎实推进城乡绿化一体化进程。加强海洋环境监测，有效改善海域生态环境。扎实推进殡葬改革，加大对“三沿五区”乱建坟墓的整治力度。强化岸线资源保护，实行严格的岸线管制措施。严格控污减排，深化石板材、造纸、化工、印染、合成革等重点行业和工业园区、企业集中区等重点区域整治，继续推进“两江三溪”和乡（镇）以上饮用水源保护区环境综合整治。

3、坚持教育优先发展。加大各级财政对教育投入力度，重视师资培养，提高教师队伍素质。加快“双高普九”进程，巩固义务教育成果，确保福安、福鼎、霞浦、柘荣实现“双高普九”目标。完善中小学布局，促进义务教育均衡发展。继续实施中小学校舍安全工程、寄宿制学校建设和城镇中小学改扩建项目，启动宁德师范学院附小新校区和市特教学校建设。积极发展学前教育，合理规划布局公办幼儿园，积极扶持、规范管理民办幼儿园。强化发展基础教育，着力推进优质高中建设。加快发展职业教育，优化整合中职学校资源，加强示范专业和实训基地建设。支持发展高等教育，加强宁德师范学院配套建设，加快推进宁德职业技术学院新校区建设。

4、提高医疗卫生水平。深化医药卫生体制改革，实施基本药物制度，促进基本公共卫生服务逐步均等化，推进公立医院改革试点，完善市、县、乡三级疾病预防控制体系和突发公共卫生事件应急机制、医疗救治体系、卫生监督体系。加快推进市医院迁建工程、市精神卫生中心病房楼建设，开工建设市中心血站、市医疗废弃物处理中心。建立完善计划生育利益导向体系，综合治理出生人口性别比偏高问题，开展免费孕前优生健康检查项目试点，提高出生人口素质，稳定低生育水平。

5、推进社会管理创新。开展“六五”普法工作，提高全民法律素质。强化督查督办，做好流动人口服务管理，积极开展法律援助。关注社区管理人员和村干部待遇问题，加强农村“六大员”评聘用管，提高社区、农村的服务水平。深入开展新一轮“平安宁德”建设，健全社会治安防控体系。进一步实施依法处理信访事项“路线图”，妥善化解和处理矛盾纠纷。落实生产安全、交通安全、校园安全、消防安全、社会安全、食品安全的管理职能和监管责任，健全突发公共事件预警和应急处置机制。

（五）繁荣大文化，提升环三软实力

加强公民道德建设和青少年思想道德教育，扎实开展群众性精神文明创建活动。进一步深化文明城市创建活动，力争年内有3个县进入全省创建文明城市先进行列、5个县进入全市创建文明城市先进行列。推进公共文化体系建设，加快文化馆、文化广场等公共文化基础设施建设，加快市妇女儿童活动中心、图书馆建设，推进海峡两岸民族文体中心、市博物馆、大剧院前期工作，继续推进农家书屋、乡镇综合文化站、文化信息共享工程等惠民工程建设。保护和发展闽东畲族文化、宗教文化、廊桥文化、海洋文化、旅游文化和红土地文化等地方特色文化以及非物质文化遗产，打造一批代表宁德形象的文化品牌。继续做好第三次全国文物普查内业工作。继续抓好广播电视村村通和农村电影放映工程建设工作。重视发展体育事业，建设市老年人体育活动中心、县（市、区）青少年校外体育活动中心，积极开展全民健身活动。大力扶持福安银器、柘荣剪纸、寿宁乌金紫砂陶、畲族银器工艺等闽东特色文化产业发展，打造区域品牌，促进特色文化产业形成规模。加快发展新闻出版、文化旅游、演艺娱乐、广告会展等文化创意产业，着力培育一批中小型文化创意企业。

在抓好五项重点工作的同时，要高度重视其他各项事业发展。全面启动老区村跨越发展工程，认真落实库区移民后期扶持政策，支持“老、少、边、岛”地区加快发展，组织好对口帮扶，完成对口支援任务。全力争创“全国双拥模范城”三连冠。加强国防教育，抓好民兵、预备役部队建设，扎实做好国防动员工作。深入开展拥军优属和军民共建活动，进一步密切军政军民关系。全面落实党的民族宗教政策，依法管理宗教事务，切实维护民族团结。认真做好老干部工作，扎实推进居家（机构）养老服务，积极做好新形势下的侨务、外事工作，继续做好人事、编制、审计、统计、档案、地震、地方志、农机、水文、气象、关心下一代等工作。

四、强化措施，保障2011年目标任务全面完成

（一）实施“五大战役”，大干开局之年

打好重点项目建设战役。实施“重点项目攻坚年”，突出抓好一批工业、能源、交通、城建环保和社会事业项目建设，全市计划安排重点建设项目170个，年度计划投资236亿元，占城镇项目投资计划的70%以上。其中，在建项目116个，年度计划投资221亿元；预备项目33个，年度计划投资15亿元；安排市重点推进前期项目21个；力争新开工26个、竣工36个。打好新增长区域发展战役。突出抓好溪南、漳湾、赛江、沙埕等四大工业片区和山区重点工业片区建设，年度完成投资170亿元。打好城市建设战役。实施“城市功能提升年”，落实公交优先，改造完成主次干道沥青路面，加快供水、供气、排水和电线下地工程建设，推进城市公厕、停车场、立体绿化和公园建设，做到步行10-15分钟就能看到公厕和绿地；创建一批物业管理示范小区，建设一批宜居城市综合体。打好小城镇改革发展战役。完善2个省级、17个市级小城镇综合改革试点的规划体系，新增2个省级试点镇；加快推进基础设施和公共服务设施建设，力争省、市级试点镇项目投资额分别增长200%、50%以上；发挥农村土地整治和城乡建设用地增减挂钩、旧城镇旧厂房旧村庄改造等政策效应，加快城镇户籍制度改革，促进试点小城镇产业和人口集聚。打好民生工程战役。扎实开展“民生工程推进年”活动，进一步加大财力保障、政策保障、工作保障力度，统筹推进以保障和改善民生为重点的社会建设，切实解决影响民生的突出问题，增强民生建设普惠性。

（二）推进品牌带动，增强发展竞争力

依托现有获得中国驰名商标的企业，引导关联企业深度分工协作，整合行业资本、技术、人才资源，形成一批主业突出、产业关联度大、带动效应明显、以行业标志性知名品牌企业为龙头的企业体系，做大做强品牌效益。加强行业共有品牌的保护、提升，扩大福安“坦洋工夫”、“福鼎白茶”、“古田银耳”、“柘荣太子参”等驰名商标和地理标志产品影响力，打造“金闽红”、“太姥银针”等区域品牌，宣传推介蕉城天山绿茶、寿宁高山乌龙茶、周宁官司云雾茶、霞浦海带和紫菜等新品牌。重视市场拉动，鼓励品牌企业在国内外设立代理机构、开设专卖店、加工基地，加大自主品牌市场销售力度，增强品牌市场影响力。建立扶持品牌的支撑体系，完善品牌的培育、激励、推介、保护工作机制，继续实行创牌重奖政策，力争新增中国驰名商标2个以上。

（三）深化改革创新，增强发展活力

持续推进市、县政府机构改革，深化乡镇机构改革。完善国有资产监督管理制度，健全国有资本收益收缴、经营预算制度。积极探索建立以市国投公司为主，新设立交通、旅游、港口和城市建设投资公司等多元的国有资产融资平台体系。积极引导和鼓励社会投资，引入市场机制，采用项目捆绑招标、BT 、BOT、代建制和特许经营权转让等多种方式，鼓励和引导民间资本参与国有企业改革和基础设施、公用事业建设。落实支持民营经济发展的各项政策措施，实施“回归工程”，鼓励海内外乡贤回乡投资兴业。加强与开发性银行、进出口银行及商业银行的沟通合作，积极引进市外银行在我市直接设点营业，组建宁德农村商业银行。加快小额贷款公司试点工作，加强中小企业信用担保体系建设，规范融资性担保公司发展。加大企业上市推进力度，选择3－5家企业作为重点培育对象，力争有1－2家拟上市企业进入辅导期。着力提高自主创新能力，加强创新服务平台建设，加快中科院中国宁德新城镇综合改革示范区一期、福建（宁德）微软技术中心、国家级中小型电机产品监督检验中心、闽东电机国家产业集群规划和高新技术产业化基地建设，支持重点骨干企业设立重点实验室、工程技术中心，抓好“6?18”项目对接和成果转化，新认定高新技术企业10家。实施“人才强市”战略，根据产业发展需求，大力加强人才队伍建设，加快创新人才培养和引进，鼓励有条件的企业设立博士后科研流动站，积极培养和引进学术技术带头人、科技领军人才、一线创新人才和创新团队。

（四）扩大对外开放，增强发展动力

继续办好第二届海峡两岸电机电器博览会、第12届宁德投洽会，举办温州、泉州项目对接会，积极组织和参加我省在北京举办的大型民营企业产业项目洽谈会以及“5?18”、“6?18”和“9?8”等重大会展活动，力求在招大商引大项目上有新突破。加强与浙南地区、闽南地区和港澳地区的区域合作，主动承接其产业、资金、技术和人才转移。积极扩大外贸出口，完善福建省电机出口基地发展规划，加快推进省电机出口基地建设。组织企业参加国内外重要展会，积极开拓自贸区市场，巩固欧美日等传统市场，拓展东盟、拉美、非洲等新兴市场。拓展宁台交流合作，把握ECFA正式签署的有利时机，推进宁台产业对接、文化交流和人员往来。积极推动台湾水产品集散中心、台湾农民创业园的规划建设，主动承接台湾电机电器、冶金、石化产业和现代农业。完善宁德―基隆经济文化对口合作协调机制，加强两地在口岸、仓储、运输、物流、加工等相关港口配套产业方面的深度交流合作。推进福鼎点头镇、福安社口镇、霞浦三沙镇与台湾嘉义阿里山乡、南投竹山镇和台中沙鹿小区的交流合作。

（五）以“创先争优深化年”为抓手，推进依法行政，建设高效廉洁的服务型政府

1、切实提高审批效率。把提高审批效率作为改革创新的突破口，敢于运作，灵活变通，推进项目落地。推行“容缺预审”办法，对具备可研报告、选址符合规划、限制类项目的环境影响评价、土地预审、资金证明等基本要件的项目，先予以办理审批手续，做到“依法依规，程序不减，时间压缩”。创新“并联审批”方法，实行重大项目“并联会审”，采取联合审批办公会议方式，组织相关职能部门集中研究重大项目审批要素，明确审批时限，促进审批提速提质。

2、积极改进工作作风。各级政府要强化时间观念、效率观念，立足实干苦干，争时间、抢速度，推动突破促落实，确保各项目标任务按时完成。继续实行市领导挂县、县领导包乡、干部驻村任职、科技特派员等制度，加强调查研究，推广现场办公，经常深入到基层一线，深入到问题多、矛盾尖锐、工作推不开的地方，与基层干部群众一起研究问题、解决问题，推动工作落实在基层、问题解决在一线。

3、严格落实工作责任。各级领导干部要提振精神、激情创业，求真务实、真抓实干，切实提高工作效率和办事速度。要敢于负责、勇于负责，严格落实工作责任制和领导干部问责制，对工作中遇到的问题，不说“不能办”，多想“怎么办”，特别是对重大招商项目和建设项目，按照“五个一”项目运作工作机制、“六比六看”项目建设推进办法和“九个具体”项目管理办法推进项目落实，对没有按时完成的部门和责任人，严格实行问责，不能让庸懒者逍遥自在，充分体现责任对保障工作落实的严肃性和权威性。

4、加强政府廉政建设。深化机关效能建设，加强效能督查、投诉办理和行政问责，全面实行绩效管理，进一步治庸治懒。抓好公务员队伍建设，增强公务员考录工作的透明度，加强公务员的教育培训，提高公务员队伍的整体素质。推进行政职权清理、后续监管和政务公开，防止权力配置资源和不当介入经济活动。广泛开展公务员行为规范教育、理想信念教育和廉洁自律教育，健全完善惩治和预防腐败体系，落实党风廉政建设责任制，促进社会公平正义。

5、始终坚持依法行政。坚持向人大及其常委会报告工作和向人民政协通报情况，自觉接受监督，认真办好人大代表建议、意见、批评和政协提案。依法接受市人大常委会对有关政府工作部门和省以下垂直单位开展专项询问活动，支持市政协委派监督小组到政府重点部门开展民主监督试点工作。建立健全社会公示和听证制度，完善政府信息披露和民意反馈机制。密切与各民主党派、工商联、无党派人士和社会各界的联系，充分发挥工会、共青团、妇联等人民团体联系群众的桥梁纽带作用，充分听取市民的意见和建议，维护群众合法权益。规范行政决策程序，完善决策评估和决策纠错机制，使决策合法合理、行之有效。贯彻落实国务院依法行政实施纲要、加强市县政府依法行政和加强法治政府建设的意见，规范行政执法，做好行政复议工作，严格行政执法责任制和责任追究制，提高政府公信力和执行力。

各位代表！全力推进宁德科学发展、跨越发展，是时代赋予我们的重任，也是闽东人民寄予我们的重托。我们要紧密地团结在以胡锦涛为总书记的党中央周围，在省委、省政府和市委的坚强领导下，大力弘扬“滴水穿石、人一我十、力求先行”的闽东精神，凝聚全市广大干部群众的智慧和力量，坚定信心，开拓创新，扎实苦干，奋发有为，为开创“十二五”经济社会发展新局面、推进环三跨越崛起而努力奋斗！
